# Association Between Tumor Budding and Lymph Node Metastasis in Surgically Resected Colon Cancer: A Retrospective Observational Study

**DOI:** 10.3390/medicina62101861

**Published:** 2026-09-25

**Authors:** Alisina Bulut, Muhammed İkbal Akın, Beril Kara Esen, Niyaz Shadmanov, Zeynep Sonmez, Suha Goksel, Oktar Asoglu

**Affiliations:** 1 Department of General Surgery, Faculty of Medicine, Marmara University, 34854 Istanbul, Turkey; 2 Department of Gastrointestinal Surgery, Koşuyolu High Specialization Education and Research Hospital, University of Health Sciences, 34846 Istanbul, Turkey; 3 Esenler District Health Directorate, Republic of Türkiye Ministry of Health, 34225 Istanbul, Turkey; 4 General Surgery, Bogazici Academy for Clinical Sciences, 34357 Istanbul, Turkey; 5 School of Medicine, City University of New York, New York, NY 10031, USA; 6 Department of Pathology, Acıbadem Maslak Hospital, 34457 Istanbul, Turkey

**Keywords:** colon cancer, lymph node metastasis, prognostic marker, tumor budding

## Abstract

*Background and Objectives*: Tumor budding (TB) has emerged as an important histopathological parameter associated with aggressive tumor behavior in colorectal cancer. Increasing evidence has supported its prognostic relevance, particularly regarding lymph node metastasis and adverse pathological features. This study aimed to evaluate whether the established association between TB and lymph node metastasis could be reproduced in our colon cancer cohort and to examine its relationship with other clinicopathological features. *Materials and Methods*: This retrospective observational study included 136 patients who underwent surgery for colon cancer and whose pathology reports included information on TB. TB was categorized as Bd0, Bd1, Bd2, and Bd3 to evaluate its association with lymph node positivity. The investigation also uncovered the associations between TB and several prognostic indicators, such as T stage, perineural invasion, venous invasion, tumor size, lymphatic invasion and lymph node ratio. *Results*: Higher TB grade was associated with lymph node positivity in the four-tier analysis (*p* = 0.012), as well as with lymphatic and venous invasion. However, the association between TB grade and lymph node positivity did not reach statistical significance using the standard ITBCC three-tier classification (*p* = 0.058). In the primary parsimonious multivariable model, Bd1 was associated with higher odds of lymph node positivity compared with Bd0 (aOR 3.36, 95% CI 1.02–11.02), whereas the estimates for Bd2 (aOR 3.36, 95% CI 0.96–11.75) and Bd3 (aOR 2.76, 95% CI 0.82–9.30) did not reach statistical significance. *Conclusions*: Higher TB grades were associated with lymph node positivity and other adverse pathological features in unadjusted analyses. In the parsimonious multivariable model, the association with lymph node positivity remained significant only for Bd1, while the estimates for Bd2 and Bd3 did not reach statistical significance. These findings should be interpreted cautiously given the limited sample size and the non-significant association observed using the standard ITBCC three-tier classification.

## 1. Introduction

On a global scale, colorectal cancer is the third most common form of cancer and the second most significant contributor to cancer-related mortality [1]. The ‘International Union against Cancer’ states that lymphatic and venous invasion, along with TNM stage, are the essential prognostic factors. Tumor budding (TB), tumor margin configuration, perineural invasion (PNI) and tumor grade are considered additional prognostic factors [2].

TB is defined as the presence of single cells or small clusters of less than five cells at the advancing front of the tumor, and it is significantly linked with lymph node metastases, greater tumor grade, lymphovascular invasion (LVI) and PNI [3,4]. It is also known that TB increases the risk of local recurrence and distant metastasis and causes poor survival [5]. The cumulative increase in evidence regarding the prognostic value of TB has resulted in this parameter being included as a standard in pathology reports. A unified system for TB assessment and reporting was established by the 2016 International Tumor Budding Consensus Conference (ITBCC) [6].

In this context, the present study aimed to evaluate the established association between TB and lymph node metastasis in our colon cancer cohort and to examine its relationship with other clinicopathological features, including T stage, lymphatic and venous invasion, PNI, MSI status, and tumor size.

## 2. Materials and Methods

### 2.1. Study Design and Ethical Approval

This retrospective observational study included patients who underwent colectomy performed by an experienced colorectal surgeon at a tertiary university hospital between February 2017 and July 2022. Clinical and demographic data were obtained retrospectively from the institutional electronic medical records, and pathological data were obtained from the institutional pathology database. All surgical specimens were evaluated at the same institution. Approval for the study was granted by the institutional clinical research ethics committee (reference number: 09.2024.965; approval date: 4 October 2024). The study was conducted in accordance with the principles of the Declaration of Helsinki. The authors declare that there are no conflicts of interest related to this study.

### 2.2. Cohort Formation

All consecutive patients who underwent colectomy during the study period were screened for eligibility. Patients aged ≥18 years who underwent colectomy for colon adenocarcinoma and had evaluable TB data were eligible for inclusion. Patients were excluded if the pathology report was unavailable, the final pathology was benign, surgery was performed for recurrent disease or peritoneal carcinomatosis, or TB could not be evaluated because of missing information in the pathology report or treatment-related histopathological changes following neoadjuvant therapy. The sample size was not determined by special calculations; it was deemed sufficient for the number of patients who met the appropriate criteria.

### 2.3. Data Collection and Definitions

Patients without TB were referred to as Bd0; those with budding were referred to as Bd1, Bd2 and Bd3 from low to high. The lymph node ratio (LNR) was defined as the proportion of positive lymph nodes to the total number of lymph nodes harvested. The association between TB and various prognostic factors, including tumor size, lymph node status, lymphatic and venous invasion, PNI, microsatellite instability (MSI) status, and LNR, was examined. From electronic medical data, the pathological T and N staging according to the 7th edition of the American Joint Committee on Cancer (AJCC) was retrieved [7]. Demographic details were also obtained from medical records. Tumor location was determined from operative and pathological records. Tumors documented as arising in the rectosigmoid region were classified as rectosigmoid tumors. Rectal cancers were not included in the study. MSI status was obtained retrospectively from pathology reports based on immunohistochemical assessment of the mismatch repair (MMR) proteins MLH1, PMS2, MSH2, and MSH6. Preserved expression of all four proteins was classified as MSI-negative, whereas cases reported as MSI-H based on loss of MMR protein expression were classified as MSI-positive.

### 2.4. Histopathological Evaluation

Evaluation of TB was conducted according to the standards established by the 2016 ITBCC [6]. Peritumoral TB was defined as the presence of single tumor cells or small clusters of fewer than five tumor cells at the advancing tumor front [8]. All TB evaluations were performed by an experienced gastrointestinal pathologist on 4-μm-thick hematoxylin and eosin-stained sections prepared using an autostainer. TB was assessed as part of the routine histopathological evaluation of the resection specimens and was not retrospectively reassessed specifically for the present study. TB data were extracted from the corresponding pathology records. As these evaluations were conducted during routine pathological assessment, the pathologist was not formally blinded to lymph node status or other pathological findings.

In accordance with routine practice in our pathology laboratory, the entire tumor was sampled in colorectal carcinoma resection specimens, with the number of tumor blocks varying according to tumor size. All tumor-containing sections were initially examined at ×10 objective magnification to identify candidate budding hotspots at the advancing tumor front. The area showing the highest budding density was subsequently selected and tumor buds were counted at ×20 objective magnification. An Olympus BX43 microscope (Olympus Corporation, Tokyo, Japan) with an eyepiece field number of 25 mm was used for TB assessment. At ×20 objective magnification, this corresponded to a specimen-field diameter of 1.25 mm and a radius of 0.625 mm, yielding a field area of 1.227 mm^2^. To standardize the bud count to the ITBCC reference area of 0.785 mm^2^, a normalization factor of 1.563 (1.227/0.785) was applied. Accordingly, the normalized tumor bud count per 0.785 mm^2^ was calculated by dividing the observed bud count at ×20 magnification by 1.563. Tumors with no identifiable buds were categorized as Bd0. Among tumors with budding, Bd1 was defined as 1–4 buds, Bd2 as 5–9 buds, and Bd3 as ≥10 buds per 0.785 mm^2^. Thus, a four-tier categorization (Bd0–Bd3) was used in the primary analyses to distinguish tumors with complete absence of budding from those showing low-level budding.

In mucinous and medullary carcinomas, TB was assessed cautiously and only in areas where tumor buds could be reliably distinguished from histology-related artifacts. In mucinous carcinomas, budding was evaluated at the invasive front in areas showing an infiltrative intestinal-type component, while isolated tumor cells or clusters suspended within mucin pools were not counted as tumor buds. In medullary carcinomas, only unequivocal tumor buds at the invasive front were considered, avoiding areas in which prominent inflammation or tumor-cell discohesion precluded reliable distinction of true budding.

### 2.5. Outcomes of the Study

The primary objective was to evaluate the association between TB and lymph node positivity. Secondary objectives involve examining how TB correlates with prognostic factors like T stage, tumor size, venous and lymphatic invasion, PNI, and MSI status.

### 2.6. Statistical Analysis

Statistical analyses were performed using SPSS v.21 (SPSS Inc., Chicago, IL, USA). Continuous variables were compared using the Mann–Whitney U test for two-group comparisons and the Kruskal–Wallis test for comparisons across more than two groups, while categorical variables were analyzed using the chi-square or Fisher’s exact test, as appropriate. For significant overall chi-square tests, post hoc pairwise comparisons of column proportions were performed with Bonferroni adjustment. Spearman correlation analysis was used to assess associations involving non-normally distributed or ordinal variables. Univariate binary logistic regression analyses were used to evaluate the associations between TB grade and binary pathological outcomes, with results reported as odds ratios and 95% confidence intervals.

Univariate analyses were performed to identify clinicopathological factors associated with lymph node positivity. Variables with a *p*-value < 0.20 in the univariate analyses were considered candidates for the multivariable logistic regression model. To limit model complexity and reduce the risk of overfitting, clinically related or potentially redundant variables were not entered simultaneously. Accordingly, differentiation was selected instead of tumor grade, and tumor size was not additionally included because T stage was already incorporated into the model.

The multivariable logistic regression analysis was performed using complete-case analysis. Of the 136 patients, two had missing data for at least one covariate included in the model, resulting in a final model sample size of 134 patients. Multicollinearity was assessed using variance inflation factors (VIFs) and tolerance values; VIFs ranged from 1.35 to 3.34, and all tolerance values were ≥0.30. Given the possibility that lymphatic and venous invasion may represent intermediate pathological features in the pathway between tumor budding and lymph node metastasis, these variables were excluded from the primary parsimonious multivariable model. An extended model including lymphatic and venous invasion was additionally evaluated as a sensitivity analysis. Model fit was assessed using the Hosmer–Lemeshow goodness-of-fit test and Nagelkerke R^2^.

Ordinal logistic regression was used to evaluate the association between T stage and TB grade, with model assumptions and fit assessed using goodness-of-fit tests, the test of parallel lines, and model fitting information. In a separate sensitivity analysis, associations between TB grade and the main pathological outcomes were re-evaluated using the conventional ITBCC three-tier classification by combining Bd0 and Bd1 into Bd1 (0–4 buds). A two-sided *p* < 0.05 was considered statistically significant.

## 3. Results

A total of 232 consecutive patients who underwent colectomy during the study period were screened for eligibility. Three patients were excluded because pathology reports were unavailable. Of the remaining patients, 31 with benign pathology, 12 who underwent surgery for recurrent disease, and five with peritoneal carcinomatosis were excluded. Among the remaining 181 patients, TB could not be reliably evaluated in 19 because of neoadjuvant treatment-related histopathological changes, and TB information was not available in the pathology reports of 26 patients. After these exclusions, 136 patients met the eligibility criteria and were included in the final analysis (Figure 1).

Among these patients, 85 (62.5%) were male and 51 (37.5%) were female, with a median age of 59 years (50–67). The rectosigmoid (30.9%) and sigmoid colon (27.2%) were the most common tumor locations, followed by the right (17.6%), transverse (13.2%), and left colon (4.4%); synchronous tumors were present in 6.6% of patients. TB was detected in varying frequencies among patients’ pathology reports: 27 (20%) patients exhibited no tumor budding, while 39 (29%), 33 (24%), and 37 (27%) patients demonstrated Bd1, Bd2, and Bd3 classifications, respectively. Histological examination revealed diverse tumor subtypes, with adenocarcinoma being the most prevalent, 93 (68.3%), followed by cribriform comedo type 19 (14%), mucinous 19 (14%), and medullary carcinoma 5 (3.7%). A statistically significant difference was not found between TB grades and factors including age, gender, differentiation status, and grade (Table 1). Three patients (2.2%) had received neoadjuvant treatment before surgery. Stage IV disease with distant metastases was present in 19 patients (14.0%).

Regarding pathological stage, among the 135 patients with available T-stage data, 2 (1.5%) had Tis, 1 (0.7%) had T1, 14 (10.4%) had T2, 64 (47.4%) had T3, and 54 (40.0%) had T4 tumors. Among the 135 patients with available N-stage data, nodal stage was N0 in 53 (39.3%), N1 in 40 (29.6%), and N2 in 42 (31.1%) patients. The median numbers of harvested, and positive lymph nodes were 32.5 and 2.0, respectively, and more than 12 lymph nodes were harvested in all patients. Higher TB grade was positively correlated with the number of positive lymph nodes (r: 0.340, *p* < 0.001), LNR (r: 0.326, *p* < 0.001), T stage (r: 0.242, *p*: 0.005), and N stage (r: 0.285, *p* < 0.001). In contrast, no significant correlation was observed between TB grade and the total number of harvested lymph nodes (r: 0.073, *p*: 0.401) or tumor size (r: −0.056, *p*: 0.517) (Table 2).

More than half of the patients (60.3%) had positive lymph nodes, with a significant association found between lymph node positivity and TB grade (*p*: 0.012). Of the patients, 88 (65.2%) had lymphatic invasion and 87 (64.4%) had venous invasion. A significant association was detected between TB grade and both lymphatic and venous invasion (*p* < 0.001 and *p*: 0.002, respectively). Patients with Bd2 and Bd3 exhibited significantly higher rates of lymphatic invasion, venous invasion, and lymph node positivity compared to Bd0 patients. Additionally, lymphatic invasion was significantly more common in Bd3 patients compared to Bd1 patients (Table 3). In the sensitivity analysis using the conventional ITBCC three-tier classification, the association between TB grade and lymph node positivity was attenuated and became borderline but did not reach statistical significance (*p*: 0.058), compared with the significant association observed using the four-tier classification (*p*: 0.012).

PNI was present in 63 (46.7%) of patients, and there was no significant relation between PNI and TB. Compared to those with Bd0, the odds of venous invasion were 7.37 in Bd2 and 5.29 in Bd3. The odds of lymphatic invasion or lymph node positivity were also significantly higher with Bd1, Bd2 or Bd3 compared to Bd0. In addition, PNI tended to be more frequent in patients with Bd2 and Bd3 than in those with Bd0; however, this finding should be interpreted cautiously given the borderline overall statistical significance (Table 4).

Univariate analyses of clinicopathological factors associated with lymph node positivity are presented in Table 5. Lymphatic invasion, venous invasion, perineural invasion, and T stage were significantly associated with lymph node positivity. Differentiation, tumor grade, and tumor size also met the prespecified *p* < 0.20 threshold for consideration in the multivariable analysis. To avoid redundancy and excessive model complexity, differentiation was selected instead of tumor grade, and tumor size was not included because T stage was incorporated into the multivariable model (Table 5).

The primary parsimonious multivariable model included 134 patients with complete data. T4 stage was independently associated with lymph node positivity (aOR 6.01, 95% CI 1.45–24.87; *p* = 0.013). Compared with Bd0, Bd1 was associated with higher odds of lymph node positivity (aOR 3.36, 95% CI 1.02–11.02; *p* = 0.046), whereas the estimates for Bd2 (aOR 3.36, 95% CI 0.96–11.75; *p* = 0.058) and Bd3 (aOR 2.76, 95% CI 0.82–9.30; *p* = 0.102) did not reach statistical significance. The model showed acceptable fit (Hosmer–Lemeshow *p* = 0.894; Nagelkerke R^2^ = 0.318) (Table 6). In the extended sensitivity model additionally including lymphatic and venous invasion, the effect estimates for tumor budding were attenuated and the confidence intervals widened, while the overall interpretation remained cautious.

In ordinal logistic regression analysis, a one-stage increase in T stage was associated with 1.77-fold higher odds of being in a higher TB category (OR 1.77, 95% CI 1.24–2.51; *p* = 0.002). The proportional-odds assumption was satisfied (Test of Parallel Lines, *p* = 0.783), and the Nagelkerke R^2^ was 0.082.

## 4. Discussion

We investigated the relationship between TB and lymph node positivity. We also evaluated the relationship of TB with age, sex, tumor size, tumor grade, lymphatic and venous invasion, PNI and T stage. High-grade TB (Bd3) has been reported in up to 40% of colorectal cancers [9]. In our cohort, the Bd3 rate was 27%, which is consistent with the literature. The present study revealed that tumors without TB had fewer positive lymph nodes than tumors with Bd2 and Bd3. Compared to patients without TB, the odds of lymph node positivity was 4.7 times higher in patients with Bd3. Our findings are consistent with previous studies demonstrating an association between TB and lymph node metastasis [10], providing additional evidence from an independent single-center cohort. Although the conventional ITBCC classification includes tumors with 0–4 buds within the Bd1 category, we retained tumors with no identifiable budding as a separate Bd0 category in the primary analysis. This approach is supported by previous evidence suggesting that zero-budding tumors may represent a distinct subgroup within conventional Bd1 tumors [11].

Notably, the association between TB grade and lymph node positivity was significant using the four-tier classification but did not reach statistical significance with the conventional ITBCC three-tier classification (*p* = 0.058). In the primary parsimonious multivariable model, Bd1 was associated with higher odds of lymph node positivity compared with Bd0, whereas the estimates for Bd2 and Bd3 did not reach statistical significance. The absence of a clear dose–response pattern across increasing budding categories, together with the non-significant result obtained using the conventional ITBCC classification, warrants cautious interpretation of this finding. Previous studies using multivariable analyses have reported high-grade TB as an independent predictor of lymph node metastasis and recurrence-related outcomes [12,13]. In contrast, our findings did not demonstrate a consistent independent association between increasing TB grade and lymph node positivity. This discrepancy may partly reflect the relatively small sample size and the close relationship between TB and other adverse histopathological features. Furthermore, when lymphatic and venous invasion were included in an extended sensitivity model, the effect estimates for TB were attenuated and none of the TB categories remained statistically significant. Taken together, these findings suggest that the observed relationship between TB and lymph node positivity may partly overlap with other adverse pathological characteristics and should be interpreted cautiously.

Tumor size has been associated with the risk of lymph node metastasis [14]. In this study, although no significant correlation was observed between tumor size and TB grade, a one-stage increase in T stage was associated with 1.77-fold higher odds of being in a higher TB category. Although T1 tumors can be node positive and T4 tumors node negative, this apparent paradox can be explained by recent advances in tumor biology. Adenocarcinomas are not biologically identical; each tumor has its own molecular “fingerprint” shaped by its microenvironment [15]. TB, an emerging histopathological feature in colorectal cancer, appears to be an important component of this fingerprint [16]. The standardized ITBCC assessment method has facilitated the incorporation of TB into clinical risk stratification across different tumor stages, particularly in early colorectal cancer (T1/T2) cohorts where high-grade budding serves as a crucial predictor of occult lymph node metastasis and guides subsequent surgical or therapeutic decisions [17,18].

Lymph node metastasis by tumor cells spreading through lymphatic channels or venules is considered to be an early stage of colorectal cancer metastasis [19]. In the present study, lymphatic invasion was found in 65% and venous invasion in 64%. The relationship between the degree of TB and lymphatic and venous invasion was also significant and consistent with the literature [20]. Shalkamy et al. reported the lymphatic invasion rate as 45.6% and the venous invasion rate as 13.7% [21]. The literature reports a wide range of lymphovascular invasion rates, from 5.2% to 89.5%. Differences in tumor characteristics and different staining techniques used in pathological evaluation may be effective in reporting such different rates [22].

A few studies have been done on the relationship between PNI and lymph node metastasis in colon cancer, compared to the vascular infiltration of colorectal cancer [23]. The reported incidence of PNI ranges from 13.6% to 50% [22,23]. The 46% PNI positivity found in this study is consistent with the literature. Although there was no statistical difference between PNI and TB grade in the present study, PNI tended to be more frequent in patients with higher TB grades, particularly Bd2 and Bd3. To our knowledge, few studies have specifically evaluated the direct relationship between TB and PNI.

In the present study, patients were evaluated in two groups as MSI+/− and no significant relationship was found between MSI status and TB. The results of studies revealing the relationship between MSI status and TB are heterogeneous. Wright and Stewart [24] report that TB is less common in MMR-defective tumors. Other studies, in contrast, report higher rates of TB in non-MSI tumors [25,26].

This study has several limitations. Its retrospective, single-center design and relatively small sample size may have introduced selection bias and limited the generalizability of the findings. In particular, the exclusion of patients without reported or evaluable TB may have contributed to selection bias. Some clinicopathological variables had missing data, and potential confounding related to tumor stage should also be considered. In addition, TB was evaluated by a single experienced gastrointestinal pathologist; therefore, interobserver reproducibility could not be assessed. Although evaluation by an experienced pathologist may have improved consistency, it does not eliminate this limitation. Finally, the study lacked external validation and did not include oncological outcomes. Future studies incorporating disease-free, overall, and cancer-specific survival are needed to determine the independent prognostic value of TB after adjustment for established prognostic factors. However, the study also has some strengths. TB is an increasingly recognized histopathological feature in colorectal cancer. We believe that our findings provide additional data from a single-center cohort and contribute to the existing literature on the relationship between TB and pathological characteristics of colorectal cancer. To our knowledge, this study is among the relatively few studies to collectively examine the relationships between TB grade and multiple clinicopathological features, including tumor size, T stage, MSI status, LVI, PNI, and LNR. Additionally, the fact that all surgeries were performed by a highly experienced colorectal surgeon is valuable, as it ensures that the data were extracted from high-quality specimens.

## 5. Conclusions

Higher TB grades were associated with lymph node positivity and lymphovascular invasion in unadjusted analyses. In the parsimonious multivariable model, Bd1 was associated with increased odds of lymph node positivity compared with Bd0, whereas the associations for Bd2 and Bd3 did not reach statistical significance. Moreover, the association was not statistically significant when TB was categorized according to the conventional ITBCC three-tier system. Given the absence of a clear dose–response pattern, the limited sample size, and the lack of oncological outcome data, these findings should be interpreted cautiously.

## Figures and Tables

**Figure 1:**
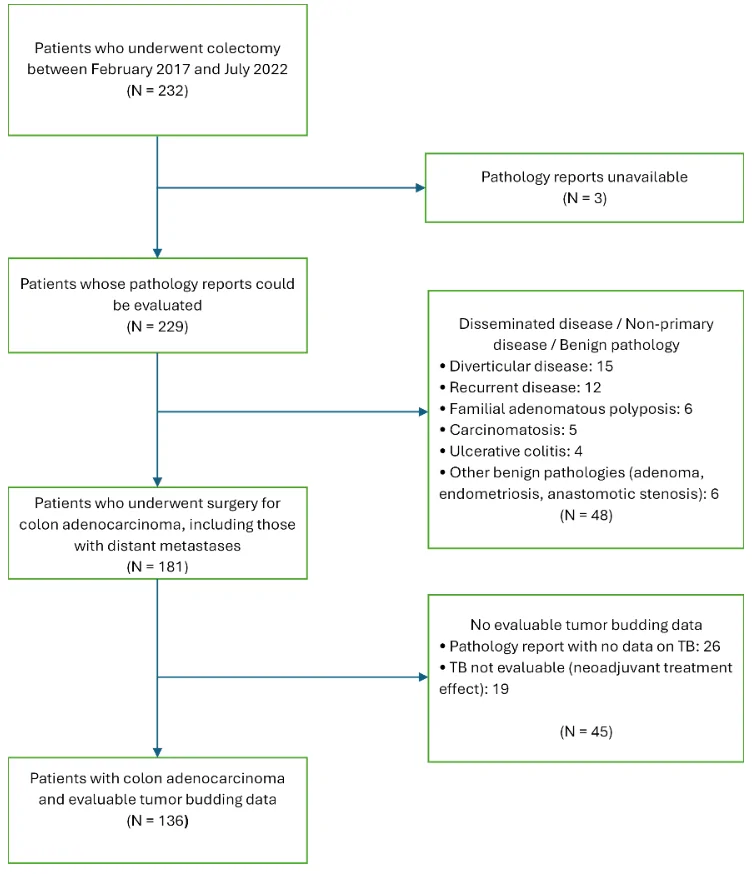
Flowchart of patient selection and study cohort formation.

**Table 1:** Association between clinicopathological variables and tumor budding.

Variable	Total (*n* = 136)	Bd0 (*n* = 27)	Bd1 (*n* = 39)	Bd2 (*n* = 33)	Bd3 (*n* = 37)	*p*
**Age, median (IQR)**	59 (50–67)	63 (47–65)	62 (54–70)	55 (49–66.5)	58 (50–69.5)	0.469 *
**Sex, *n* (%)**						0.656 **
Male	85 (62.5)	18 (66.7)	25 (64.1)	22 (66.7)	20 (54.1)	
**Histological type, *n* (%)**						
Adenocarcinoma	93 (68.3)	16 (59.3)	29 (74.3)	26 (78.8)	22 (59.5)	
Mucinous	19 (14.0)	8 (29.6)	4 (10.3)	2 (6.1)	5 (13.5)	
Comedo	19 (14.0)	3 (11.1)	4 (10.3)	5 (15.2)	7 (18.9)	
Medullary	5 (3.7)	0 (0.0)	2 (5.1)	0 (0.0)	3 (8.1)	
**Differentiation, *n* (%) †**						0.130 ***
Well	8 (5.9)	2 (7.4)	4 (10.3)	2 (6.3)	0 (0.0)	
Moderate	105 (77.8)	20 (74.1)	30 (76.9)	28 (87.5)	27 (73.0)	
Poor	22 (16.3)	5 (18.5)	5 (12.8)	2 (6.3)	10 (27.0)	
**Grade, *n* (%) ‡**						0.181 ***
Low	114 (85.1)	22 (84.6)	34 (87.2)	30 (93.8)	28 (75.7)	
High	20 (14.9)	4 (15.4)	5 (12.8)	2 (6.3)	9 (24.3)	

IQR: Interquartile Range, * Kruskal–Wallis, ** Chi-squared test, *** Fisher’s Exact Test; † Differentiation data were available for 135 patients; one observation was missing in the Bd2 group (available *n* = 32). ‡ Grade data were available for 134 patients; one observation was missing in the Bd0 group (available *n* = 26) and one in the Bd2 group (available *n* = 32). Percentages were calculated using the number of patients with available data as the denominator.

**Table 2:** Correlations between tumor budding grade and clinicopathological characteristics.

	Total	Tumor Budding Grade	
	Median (Min.–Max.)	r	*p*
**Number of lymph nodes harvested**	32.5 (13–128)	0.073	0.401
**Number of positive lymph nodes**	2 (0–49)	0.340 *	<0.001
**LNR**	0.05 (0.0–0.81)	0.326 *	<0.001
**TM size** mm	52 (10–145)	−0.056	0.517
**T stage (*n* = 135)**	*n* (%)	0.242 *	0.005
Tis	2 (1.5)		
T1	1 (0.7)		
T2	14 (10.4)		
T3	64 (47.4)		
T4	54 (40.0)		
**N stage (*n* = 135)**	*n* (%)	0.285 *	<0.001
N0	53 (39.3)		
N1	40 (29.6)		
N2	42 (31.1)		

r: Spearman’s rank correlation coefficient; LNR: lymph node ratio. * *p* < 0.05.

**Table 3:** Association between tumor budding grade and invasion patterns and lymph node status.

Variable	Missing	Total (*n* = 136)	Bd0 (*n* = 27)	Bd1 (*n* = 39)	Bd2 (*n* = 33)	Bd3 (*n* = 37)	*p*
**MSI, *n* (%)**	0 (0.0)	15 (11.0)	4 (14.8)	5 (12.8)	1 (3.0)	5 (13.5)	0.346 *
**Lymphatic invasion, *n* (%) †**	1 (0.7)	88 (65.2)	8 (29.6) ª	23 (59.0) ^a,b^	24 (75.0) ^b,c^	33 (89.2) ^c^	<0.001 **
**Venous invasion, *n* (%) †**	1 (0.7)	87 (64.4)	10 (37.0) ª	23 (59.0) ^a,b^	26 (81.3) ^b^	28 (75.7) ^b^	0.002 **
**Perineural invasion, *n* (%) †**	1 (0.7)	63 (46.7)	8 (29.6)	15 (38.5)	18 (56.3)	22 (59.5)	0.050 **
**Lymph node positivity, *n* (%)**	0 (0.0)	82 (60.3)	9 (33.3) ª	24 (61.5) ^a,b^	23 (69.7) ^b^	26 (70.3) ^b^	0.012 **

* Fisher’s Exact Test, ** Chi-squared test; † Data on lymphatic invasion, venous invasion, and perineural invasion were available for 135 patients; one observation was missing in the Bd2 group (available *n* = 32). Percentages were calculated using the number of patients with available data for each variable as the denominator. Different superscript letters indicate statistically significant differences between groups at the *p* < 0.05 level. MSI: Microsatellite instability.

**Table 4:** Univariate logistic regression analysis of associations between tumor budding grade, invasion patterns, and lymph node positivity.

Outcome	Tumor Budding Grade	OR (95% CI)	Global *p*-Value
**Venous invasion** (*n* = 135)	0	Reference	0.003
	1	2.44 (0.89–6.70)	
	2	7.37 (2.26–24.03)	
	3	5.29 (1.79–15.63)	
**Lymphatic invasion** (*n* = 135)	0	Reference	<0.001
	1	3.41 (1.20–9.70)	
	2	7.13 (2.26–22.50)	
	3	19.59 (5.20–73.81)	
**Perineural invasion** (*n* = 135)	0	Reference	0.055
	1	1.48 (0.52–4.23)	
	2	3.05 (1.04–9.01)	
	3	3.48 (1.21–10.00)	
**Lymph node positivity** (*n* = 136)	0	Reference	0.017
	1	3.20 (1.15–8.94)	
	2	4.60 (1.54–13.70)	
	3	4.73 (1.63–13.73)	

OR: Odds Ratio, CI: Confidence Interval.

**Table 5:** Univariate analysis of clinicopathological factors associated with lymph node positivity.

	Lymph Node Positivity		*p*
	No	Yes	
**Age, median (IQR)**	62 (50–67)	58 (49.25–67.75)	0.459 ***
**Sex, *n* (%)**			
Male	35 (64.8)	50 (61.0)	0.651 *
Female	19 (35.2)	32 (39.0)	
**Histological type, *n* (%)**			
Cribriform comedo-type	8 (14.8)	11 (13.4)	0.627 **
Adenocarcinoma	39 (72.2)	54 (65.9)	
Mucinous	5 (9.3)	14 (17.1)	
Medullary	2 (3.7)	3 (3.7)	
**Grade, *n* (%)**			
Low	48 (90.6)	66 (81.5)	0.149 *
High	5 (9.4)	15 (18.5)	
**Differentiation, *n* (%)**			
Poor	6 (11.1)	16 (19.8)	0.071 **
Moderate	42 (77.8)	63 (77.8)	
Well	6 (11.1)	2 (2.5)	
**Lymphatic invasion, *n* (%)**			
No	28 (51.9)	19 (23.5)	0.001 *
Yes	26 (48.1)	62 (76.5)	
**Venous invasion, *n* (%)**			
No	28 (51.9)	20 (24.7)	0.001 *
Yes	26 (48.1)	61 (75.3)	
**Perineural invasion, *n* (%)**			
No	40 (74.1)	32 (39.5)	<0.001 *
Yes	14 (25.9)	49 (60.5)	
**T stage, *n* (%)**			
≤T2	12 (22.6) ^a^	5 (6.1) ^b^	<0.001 *
T3	32 (60.4) ^a^	32 (39.0) ^b^	
T4	9 (17) ^a^	45 (54.9) ^b^	
**Tumor size, median (IQR)**	48.5 (39.25–65)	55 (40–66.25)	0.083 ***

* Chi-squared test, ** Fisher’s exact test, *** Mann–Whitney U test. Different superscript letters indicate statistically significant differences between groups.

**Table 6:** Multivariable logistic regression analysis of factors associated with lymph node positivity (*n* = 134).

Variable	Adjusted OR	95% CI	*p*-Value
**Differentiation**—Poor (Reference)			
Moderate	0.56	0.17–1.85	0.342
Well	0.16	0.02–5.68	0.096
**Perineural invasion**—No (Reference)			
Yes	2.36	0.98–5.68	0.057
**T stage**—≤T2 (Reference)			
T3	1.78	0.50–6.37	0.375
T4	6.01	1.45–24.87	0.013
**Tumor budding**—Bd0 (Reference)			
Bd1	3.36	1.02–11.02	0.046
Bd2	3.36	0.96–11.75	0.058
Bd3	2.76	0.82–9.30	0.102

Model fit: Nagelkerke R^2^ = 0.318; Hosmer–Lemeshow test, *p* = 0.894. OR: Odds ratio; CI: Confidence interval.

## Data Availability

The datasets generated and/or analyzed during the current study are available from the corresponding author on reasonable request.

## Abbreviations

The following abbreviations are used in this manuscript:

TB	Tumor Budding
PNI	Perineural invasion
LVI	Lymphovascular invasion
MSI	Microsatellite instability
LNR	lymph node ratio
